# Use of evidence from the UK Obstetric Surveillance System in policy and clinical practice: a descriptive study using citation tracking

**DOI:** 10.3310/nihropenres.14196.1

**Published:** 2026-01-08

**Authors:** Ruth Tunn, Marian Knight

**Affiliations:** 1 National Perinatal Epidemiology Unit, University of Oxford, Oxford, England, OX3 7LF, UK

**Keywords:** altmetrics, bibliometrics, clinical guidelines, impact, Overton, policy research, research evaluation, UK Obstetric Surveillance System (UKOSS)

## Abstract

**Background:**

The UK Obstetric Surveillance System (UKOSS) was established in 2005 for research into uncommon disorders of pregnancy. This study explored the use in policy and clinical guidelines of evidence generated through UKOSS during its 20-year history, in order to inform approaches to increase the impact of future studies conducted through the platform.

**Methods:**

We searched Overton (24 September 2025) and Altmetric (6 October 2025) using the DOIs of articles reporting research conducted using UKOSS to identify citations in clinical guidelines and policy documents. We summarised citation frequency and characteristics of citing documents and organisations, and calculated the time between article publication and citation. For the most highly-cited articles, we explored the context of citations in a series of case studies.

**Results:**

A total of 308 unique citations of the 96 articles reporting UKOSS studies were detected, and 62/96 articles (65%) were cited in at least one policy document or clinical guideline. A third of citations were by organisations based in the UK and Ireland, and citations were roughly evenly split between clinical guidelines (53%) and policy publications (46%). The median delay between publication of an article and citation in policy documents/guidelines was 220.8 weeks (range 0.3–904.7 weeks). The context of citations varied, and included providing rationale for specific clinical recommendations such as prioritisation of pregnant women for vaccinations and management of the conditions investigated; supplying evidence for equality guidance and impact assessment; and providing more general background on risk, burden, and/or outcomes.

**Conclusions:**

Evidence from studies conducted via the UKOSS platform has been used extensively in policy documents and clinical guidance in the UK and globally. Consideration should be given to how to speed up knowledge translation to allow pregnant women and their babies to benefit from evidence-based policies and practice with minimum delays.

## Plain language summary

The UK Obstetric Surveillance System (UKOSS) involves a network of reporting staff based across all UK consultant-led maternity units. These reporters record anonymous information about all pregnant women who experience certain uncommon conditions during pregnancy. This provides strong, nationwide evidence about conditions that are otherwise difficult to study because they are so uncommon. We wanted to understand how this evidence has been used in clinical practice and healthcare policy.

In the 20 years since UKOSS was founded, 96 articles have been published that used UKOSS to collect data. We used online databases to find where these articles have been mentioned in two types of documents: policy documents, such as reports written by government departments and thinktanks; and clinical guidelines that medical staff use to decide how to treat patients.

About two-thirds of UKOSS articles had been mentioned at least once in a policy document or clinical guideline. On average, there was a gap of about four years between the article being published and the results being cited, which is a longer delay than we would like. However, the results of older studies are also still being used up to 17 years after the articles were published. This suggests UKOSS evidence is meeting an important need for information that users cannot easily get from other places.

The evidence was used in many different ways. For example, one study looked at pregnant women who ended up in hospital with Covid-19. This study’s results led to several countries making pregnant women a priority group for vaccination. NHS England also introduced measures to protect pregnant black and minority ethnic women, who had a particularly high risk of serious illness.

Going forward, we want UKOSS evidence to be used even more often, widely, and quickly so that more mothers, babies and families can benefit sooner.

## Background

The UK Obstetric Surveillance System (UKOSS) was established in 2005 as a single, nationwide platform for research into uncommon disorders of pregnancy. UKOSS gathers data to describe the incidence, diagnosis, management, and outcomes of these conditions (typically with an expected incidence of fewer than one in 2000 births) occurring during pregnancy ^1, 2^.

Because they are uncommon, such conditions are difficult to study: a large study population is required to generate meaningful data. Clinical practice therefore typically lacks a robust evidence base. However, while individually uncommon, the collective burden of such conditions is considerable, both to affected women and to healthcare systems. As a nationwide data collection system, UKOSS covers a large enough population to make the study of uncommon conditions possible ^2^.

Clinicians and other researchers can propose conditions for study using the UKOSS platform, meaning that the choice of conditions investigated is primarily driven by clinical need for evidence ^1^. The UKOSS steering committee reviews proposals and assists in shaping a data collection form that can be completed from routinely collected data and medical notes.

We are aware from stakeholder feedback of changes to practice resulting from evidence generated through UKOSS. Perhaps the most notable recent example of these occurred during the SARS-CoV-2019 pandemic, when a UKOSS study identified that pregnant women from Black and minority ethnic groups were at substantially higher risk of severe COVID-19 requiring hospitalisation compared with white pregnant women ^3^. This led to guidance being issued to all maternity units in England advising four actions aimed at minimizing this additional risk ^4^. Foreshadowing this, a decade earlier, evidence generated through UKOSS during the 2009 H1N1 influenza pandemic underpinned recognition of pregnant women as a vulnerable group and their prioritization for early antiviral treatment ^5^. UK vaccination policy was also revised to recommend annual seasonal influenza vaccination for pregnant women ^6^.

However, there have been no systematic attempts to track the extent of the use of UKOSS evidence in policy and clinical guidance, or to evaluate who is using the evidence, and when, where and how. The emerging online platforms Overton ^7^ and Altmetric ^8^ aim to facilitate exploration of such questions about research use by tracking citations of published research articles in policy documents and clinical guidelines ^9^. We previously evaluated these resources as a means of understanding the use of research generated through the NIHR Policy Research Unit in Maternal and Neonatal Health and Care ^10^. We found that although citation tracking cannot provide a comprehensive accounting of research impact, it can provide informative insights into the relevance and reach of evidence, and when and how research is used by policymakers.

The aim of this study was to use Overton and Altmetric to explore, via citation metrics and case studies of highly cited articles, the use in policy and clinical guidelines of evidence generated through UKOSS in the 20 years since the platform’s inception, in order to inform ways to improve the impact of the research.

## Methods

### Patient and Public Involvement

Patients and the public were not involved in the design of this study. Feedback on the findings was provided by the UKOSS steering group, which includes patient representatives, prior to reporting.

### Identification of articles reporting UKOSS studies

The UKOSS coordination team maintains a list of articles published on the basis of studies undertaken using the UKOSS platform. We used this list as the main source of articles for this study. We also searched PubMed (all fields) using the query ‘UKOSS OR “UK Obstetric Surveillance System”’ to identify any relevant articles that were missing from the internal list. This search was run on 9 December 2024 and updated on 24 September 2025.

We extracted articles in the internal list and the results of the PubMed search to EndNote and excluded duplicates. One author (RT) screened the titles and abstracts and, if necessary, the full texts to identify studies that used the UKOSS platform (alone or in combination with other surveillance systems) to investigate disorders of pregnancy. We excluded the following:

- Editorials commenting on UKOSS or UKOSS studies/articles that explained the UKOSS methodology without presenting new data
- Studies that did not use UKOSS to collect data (e.g., those that used UKOSS definitions or used UKOSS as a model system)
- Studies that used published UKOSS data in comparisons, but were not undertaken through UKOSS
- Articles in which UKOSS was mentioned only in declarations of interest
- Articles in which UKOSS was not mentioned

### Identification of citations of UKOSS articles in policy documents and clinical guidelines

The proprietary research tools Overton ^7^ and Altmetric ^8^ were used under institutional licence for this study. The rationales for methodological decisions are discussed in more detail elsewhere ^10^. We searched Overton and Altmetric using the digital object identifiers (DOIs) of the identified UKOSS articles. These searches were conducted on 9 (Altmetric) and 13 (Overton) December 2024 and updated on 24 September 2025 (Overton) and 6 October 2025 (Altmetric). We filtered the Altmetric search results to return citations in ‘policy sources’ and ‘clinical guidelines’ using the platform’s classification of source type. We extracted the following information, as provided by the search platform, about documents identified by the searches: bibliographic details of the cited article (title, DOI); type and country of the citing organisation; type of citing document, and date of citation. We obtained from publishers’ websites the dates that the cited articles were first made available online.

One author (RT) screened the full text of all policy documents and clinical guidelines returned by the search and extracted the text where the UKOSS article was cited. We excluded the following:

- Documents that cited the UKOSS study on a list of excluded sources
- Academic journal articles
- Cochrane reviews
- Investigators’ brochures
- MBRRACE-UK (Mothers and Babies: Reducing Risk through Audits and Confidential Enquiries across the UK) reports of maternal confidential enquiries
- Documents that were found to not actually cite a UKOSS article
- Documents that could not be obtained in full to verify the context of the citation

The rationale for excluding MBRRACE-UK confidential enquiry reports was to obtain a conservative count of policy and guideline citations. Although the MBRRACE-UK confidential enquiries present policy recommendations, they are led by the same institution that hosts UKOSS (the National Perinatal Epidemiology Unit at the University of Oxford) and the two workstreams inform each other. This exclusion criterion avoided any over-inflation of citation counts owing to “self-citation”.

For the purposes of counting “unique” citations, we merged the following types of overlapping documents to count as a single citation:

- Translations of a document into multiple languages
- The same document published by a single organisation in multiple locations
- Documents co-authored by multiple organisations and published by more than one of the author organisations
- Multiple editions or versions of the same document
- Catalogue records for a document and the document itself
- Simultaneous publication of a clinical guideline in multiple journals

We counted multiple references to an article in a single policy document or guideline as one citation, and references to multiple UKOSS articles in the same document as one citation per article.

The number of onward citations in further policy documents of the documents that cited the original research was extracted from the Overton search output before merging overlapping documents.

### Analysis

We used MS Excel for all analyses. The number of unique citations per article is summarised as median and range. Publication latency was calculated as the time from first availability of an article online, as reported on the publisher’s website, to the date the article was cited, as reported by Overton or Altmetric. Where overlapping versions of citing documents were merged to count as a single citation, the publication date of the earliest-published citing document version was used for latency calculations. Geographic distribution of citations was visualised using Datawrapper ( https://app.datawrapper.de/).

### Case studies

We identified the five articles with the largest number of citations in policy documents and guidelines and provide a descriptive analysis of citation context. We also searched Altmetric by article DOI for mentions of these articles in the mainstream media and describe coverage in prominent outlets.

## Results

**Figure 1.**
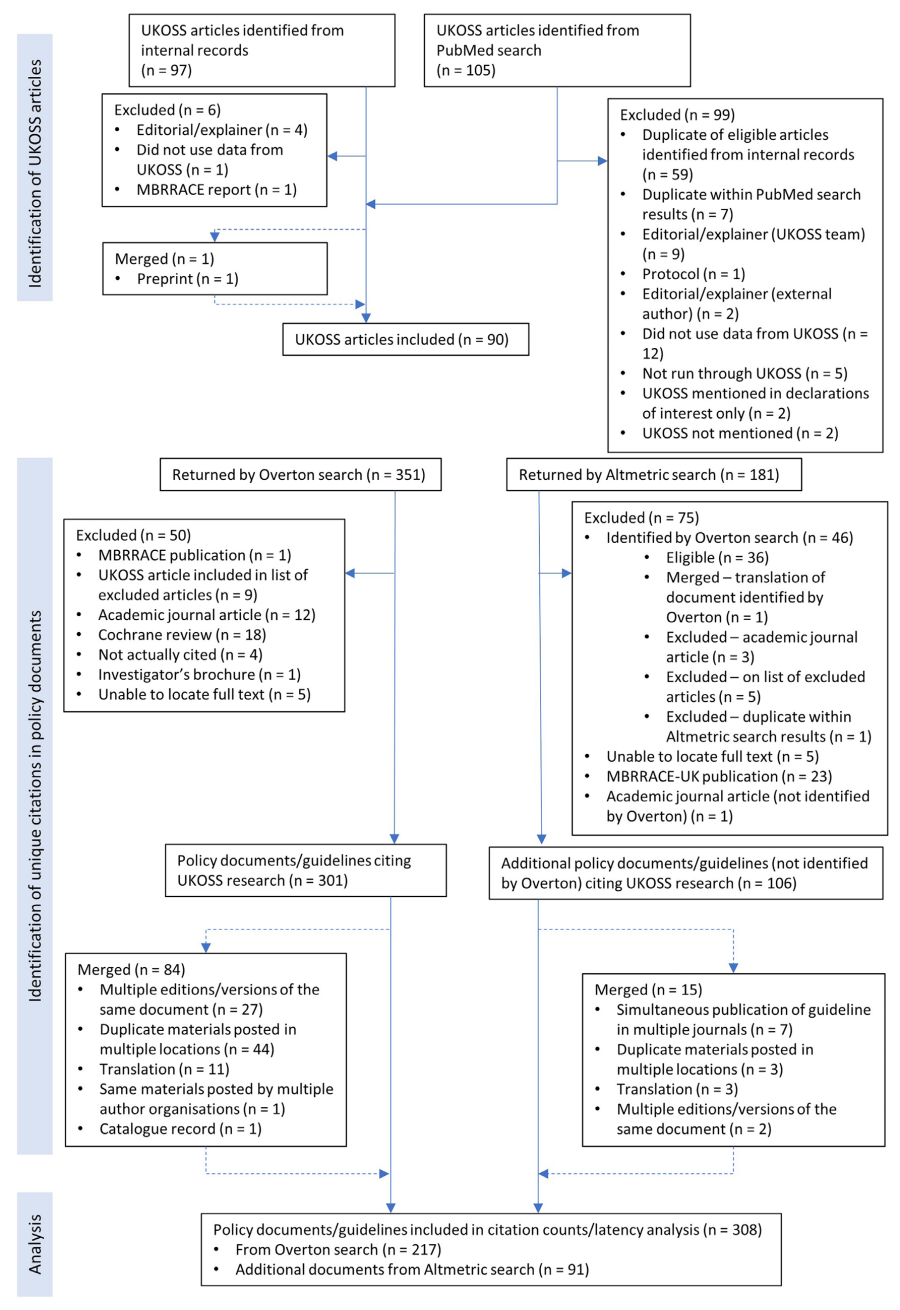
Study flow diagram. MBRRACE, Mothers and Babies: Reducing Risk through Audits and Confidential Enquiries across the UK; UKOSS, United Kingdom Obstetrics Surveillance System.

We identified 96 published articles that used the UKOSS platform to investigate disorders of pregnancy ^3, 5, 11– 104^. Searching Overton and Altmetric by the DOIs of these articles yielded 308 unique citations after exclusion of ineligible citations and merging overlapping and duplicate documents ( Figure 1). Of these, 180 (58%) were identified only by Overton, 91 (30%) were identified only by Altmetric, and 37 (12%) were returned by both tools.

**Figure 2.**
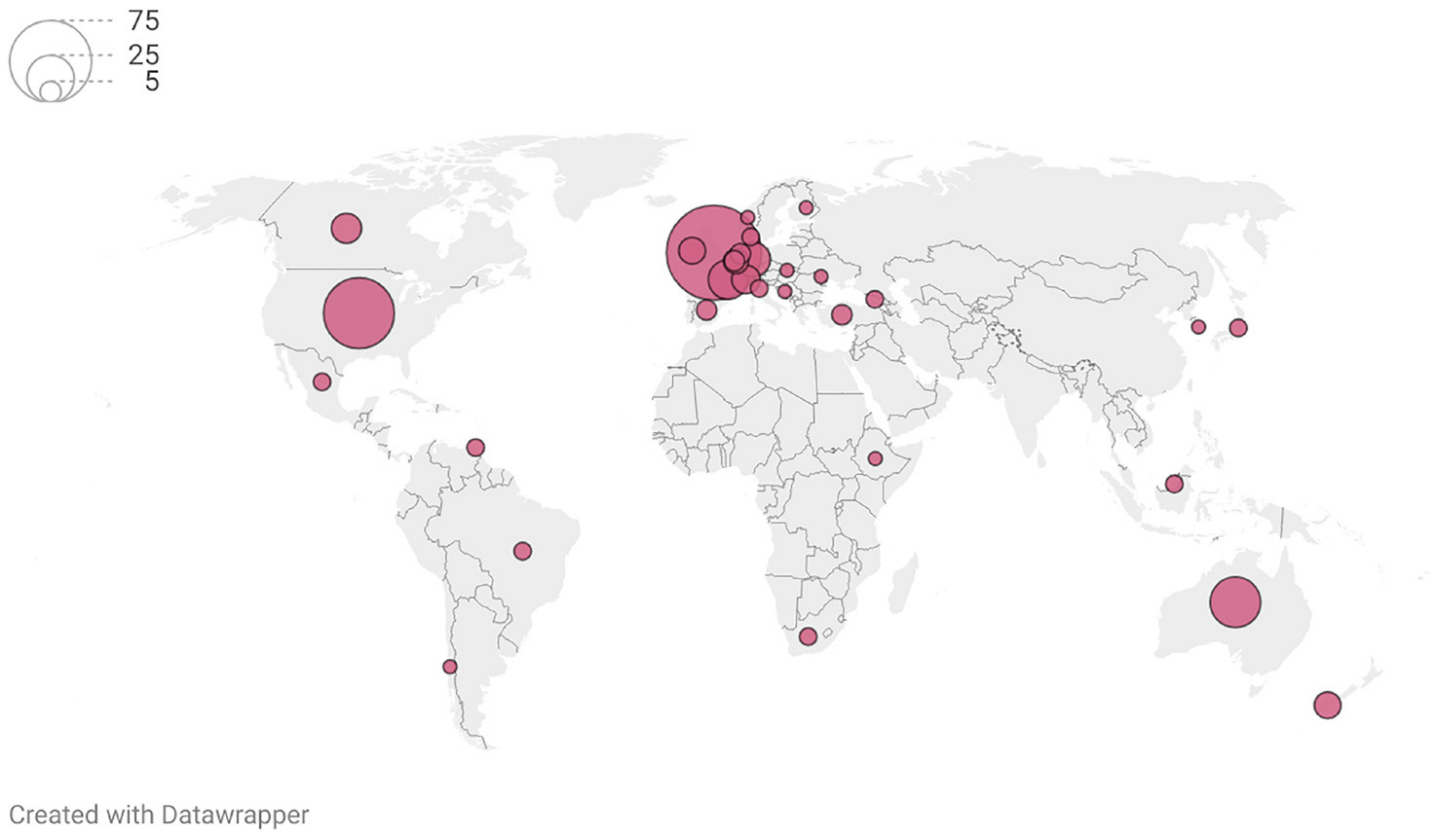
Geographic distribution of policy citations of UK Obstetrics Surveillance System evidence. Policy citations by intergovernmental organisations not visualized (n=31). Citations by European Union organisations localised to Brussels.

Of the 96 articles reporting UKOSS studies, 62 (65%) were cited in at least one policy document or clinical guideline. Characteristics of citing documents and organisations are presented in Table 1. A third of citations were by organisations based in the UK and Ireland, and organisations based in other high-income and majority English-speaking countries provided the majority of the remaining two thirds of citations. Figure 2 shows the geographic distribution of citations. The types of citing document were roughly evenly split between clinical guidelines (53%) and policy publications (46%) ( Table 1).

**Table 1.** Characteristics of policy citations of UKOSS evidence.

	Primary UKOSS studies (n = 96)
**Total number of unique policy citations**	308
**Citations per article (median, range)**	2 (0–50)
**Number of articles with ≥1 citation**	62 (65%)
**Type of citing organisation**	
Government	162 (53%)
Intergovernmental organisation	31 (10%)
Clinical organisation *	75 (24%)
Think tank	10 (3%)
Other **	30 (10%)
**Type of citing document**	
Clinical guidance	162 (53%)
Policy publication	142 (46%)
Working paper	4 (1%)
**Country of citing organisation**	
United Kingdom	101 (33%)
Ireland	6 (2%)
Other Europe/EU	56 (18%)
North America	62 (20%)
Australasia	32 (10%)
Eurasia ***	5 (2%)
Asia	5 (2%)
Central and South America	7 (2%)
Africa	3 (1%)
International organisation	31 (10%)
**Latency from article publication to ** **citation in policy document (weeks) ** **(median, range)**	220.8 (0.3–904.7)
**Total number of onward policy citations ** **(including by same organisation)**	1721
**Total number of onward policy citations ** **by different policy organisations**	1077

*e.g., clinical college, society, association

**e.g., academic institute, arms-length organisation, policy observatory, charity

***Georgia, Turkey

**Figure 3.**
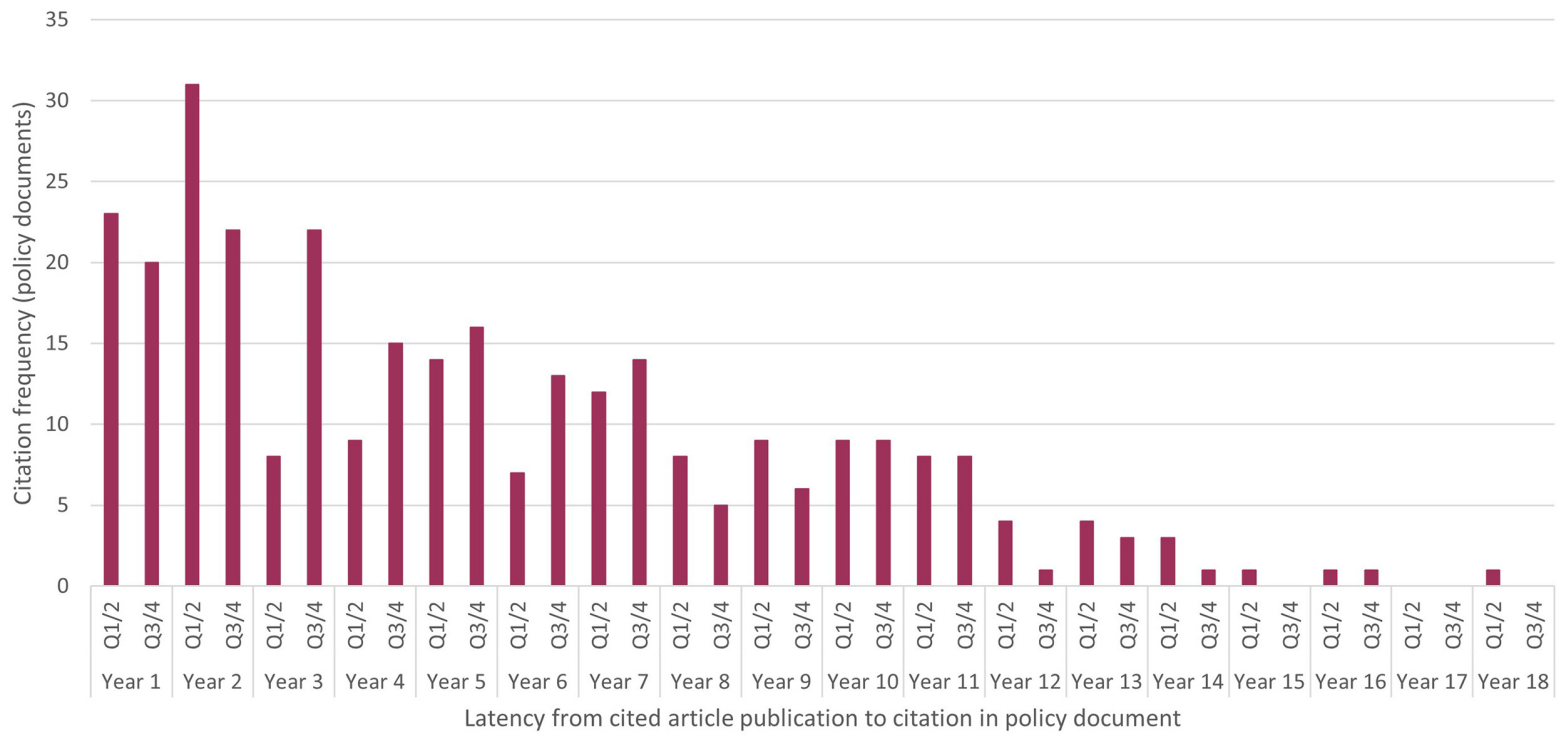
Latency from UK Obstetrics Surveillance System article publication to policy citation. The x-axis denotes the age of articles at the time of citation, with time divided into 6-month blocks (Q1/2 and Q3/4).

**Figure 4.**
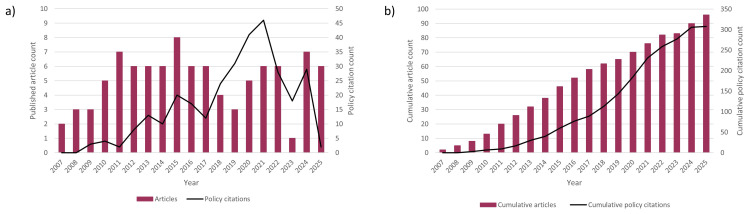
UK Obstetrics Surveillance System publications and policy citations over time. UK Obstetrics Surveillance System research article publication count and frequency of citation in policy documents **a**) year by year and **b**) cumulatively.

The median delay between publication of an article and citation in policy documents/guidelines was a little over 4 years (220.8 weeks, range 0.3–904.7 weeks), and the modal delay fell within the first half of the second year after publication ( Figure 3). Figure 4 presents the pattern of article publication and citation in policy documents and guidelines over time since the first studies using the UKOSS platform were published in 2007.

### Case study 1: Characteristics and outcomes of pregnant women admitted to hospital with confirmed SARS-CoV-2 infection in UK: national population based cohort study

The UKOSS article that was most highly cited in policy and guideline documents reported surveillance of pregnant women hospitalised with SARS-CoV-2 ^3^, with citations in 50 documents. Citations of this article were comparatively rapid: the median delay between article publication and citation in policy/guidelines was under a year (39.3 weeks, vs just under 5 years [257.7 weeks] for citations of all other articles combined) and over a third (17/50) of citations occurred within 6 months of the article being published (vs 2% [6/258] for all other articles). The most rapid citation, in an evidence summary published by the Government of New South Wales, Australia ^105^, came 2 days after the article was published.

The context of citations varied. In many cases, the evidence was used to provide background on risk, burden and/or outcomes. Other uses of the evidence had potential for more direct impact, both in the UK and globally; examples are presented in Table 2. A WHO-authored “Roadmap for research on maternal and perinatal health in the context of epidemic threats” cited UKOSS as an example of a network that allowed researchers to “ *leverage existing protocols, human and financial resources, and infrastructure to rapidly generate epidemiological data*” during the Covid-19 pandemic ^106^.

**Table 2.** Context of citations in policy documents and guidelines of the article, *“Characteristics and outcomes of pregnant women admitted to hospital with confirmed SARS-CoV-2 infection in UK: national population based cohort study”* ^3^.

Document type	Citation context	Quoted document text for which article is cited as supporting evidence	Reference
Equality impact assessment of Covid-19 lockdown policies in England	Evidence supporting recommendations around lockdowns, return to work, and work from home, with consideration of elevated risk for Black and minority ethnic pregnant women.	“ *Pregnant women are more at risk if they have underlying conditions or if they are in their third trimester of pregnancy. We consider the health benefits of social distancing for this group of pregnant women counterbalance the negative impacts"* *“Pregnant members of staff, being a clinically vulnerable group, may need adjustments, or may feel unable to return to work at the same time as their colleagues. This may be particularly pertinent for pregnant BAME members of staff as a recent study found that 56% of pregnant women admitted to hospital with coronavirus from 1 March to 14 April were from a BAME background. Clinically vulnerable individuals, which includes those who are pregnant, have been advised to take extra care in observing social distancing and should work from home”*	107
Equity and equality guidance for local maternity systems issued by NHS England	Evidence underpinning the Covid-19 recommended “four actions”	“ *Rationale and implementation: COVID-19 four actions: 56% of the pregnant women admitted to hospital with COVID-19 were from ethnic minority groups, even though they only make up a quarter of those giving birth in England and Wales. Maternity units in England were asked to take four actions to minimise the additional risk of COVID-19 to pregnant women and their babies from ethnic minorities:* *1. Increase support for at-risk pregnant women – for example, make sure clinicians have a lower threshold to review, admit and consider multidisciplinary escalation in women from ethnic minority groups.* *2. Reach out and reassure pregnant BAME women with tailored communications.* *3. Ensure hospitals discuss vitamins, supplements and nutrition in pregnancy with all women. Women low in vitamin D may be more vulnerable to coronavirus so women with darker skin or those who always cover their skin when outside may be at particular risk of vitamin D insufficiency and should consider taking a daily supplement of vitamin D all year. Folic acid can help prevent certain birth defects, including spina bifida; it’s recommended that women take a 400 micrograms folic acid tablet every day before pregnancy and until 12 weeks of pregnancy.* *4. Ensure all providers record on maternity information systems the ethnicity of every woman, as well as other risk factors, such as living in a deprived area (postcode), co-morbidities, BMI and aged 35 years or over, to identify those most at risk of poor outcomes*”	108
Written evidence to the UK government from the Equality and Human Rights Commission, Britain’s independent equality and human rights regulator	Recommendations around hygiene, social distancing, and early release for pregnant women in prison, with emphasis on the elevated risk for Black and minority ethnic women	“ *Pregnant women in prison may be at heightened risk during the pandemic. There are particular concerns for pregnant women from Black and other ethnic minority groups, who have been admitted to hospital with COVID-19 at disproportionately high rates…* *To protect the rights and health of women in prison and their children we recommend that the UK Government:…* - *Expedites appropriate releases from women’s prisons, prioritising those at heightened risk of harm. This includes those who are pregnant or have new babies, ethnic minority women and those with underlying health conditions…* - *Takes urgent steps to ensure social distancing and good hygiene can be maintained to protect the right to health of pregnant women and new mothers, where it is not possible to release these women from prison.*”	109
National and regional vaccination guidelines in Malaysia and Spain (Basque Autonomous Community)	Rationale for vaccinating pregnant women	“ *Severe illness appears to be more common in the second and third trimester. In the UKOSS study, most women were hospitalized in their third trimester or peripartum (n = 342, 81%). The median gestational age at hospital admission was 34+0 weeks of gestation (interquartile range [IQR] 29–38 weeks)…Thus, vaccinating pregnant mothers with identifiable risk factors…reduces maternal morbidity and mortality”* [Translated via translate.google.co.uk] *“Pregnant women should be encouraged to be fully vaccinated during the period of highest risk of complications from COVID-19 infection (late 2nd and 3rd trimesters of pregnancy).”*	110 111
Review of perinatal safety produced by NHS England	Rationale for priorities. The document sets out responsibilities for “board-level perinatal safety champions” to address its recommendations.	*“The 2020 MBRRACE-UK and UKOSS reports showed that 55% of pregnant women admitted to hospital with COVID-19 were from a Black, Asian or Minority Ethnic (BAME) background. Improving care for these women must be an absolute priority for maternity services across England.”*	112
Evidence update from the Africa Centres for Disease Control and Prevention	Support for social distancing in later pregnancy	“ *Findings indicate that most pregnant women admitted to hospital with SARS-CoV-2 infection were in the late second or third trimester, supporting guidance for continued social distancing measures in later pregnancy*.”	113
Evidence submitted to Japan’s Health and Science Council, Vaccination Basic Policy Subcommittee	Support for recommendation to prioritise pregnant women for vaccination	[Translated via translate.google.co.uk] “ *Top priority criteria: Pregnant women 12 weeks or over... UK Pregnancy Surveillance has reported a higher rate of hospitalization requiring respiratory support among pregnant women (41/427 cases: 9.6%).*”	114
Detailed report to an NHS Foundation Trust on “Maternity Governance – Quality & Safety”, focusing on summarising the UKOSS report and relating it to local context	The UKOSS report is the focus of the document. The document includes a section on “Guidelines and Policies” in response to the UKOSS findings of ethnic disparities.	“ *A number of specific policies, SOPs and guidelines were put in place to manage the Covid risk whilst continuing to manage a safe service. This guidance included managing the service in a different way. The changes to services are outlined in Appendix 1…A letter explaining the increased risk of women in the BAME group has been given to relevant women.* *More work is being carried out on specific additional information and advice required for women from the BAME background and for women with other co-morbidities associated with higher risk of complications of Covid.* *This work is linked to the new and existing recommendations from MBRRACE which can be found in a separate document*.”	115

Another document that cited the study was the minutes of an NHS Trust Board of Directors’ meeting along with appended reports and action plans ^116^. This revealed that “ *the UKOSS report was not discussed by the Trust's Maternity and Neonatal Safety Champion Group until 18th December 2020, thus missing the 30th November deadline* [specified in the safety action plan]”. The report reflected on the need for improved systems and processes to ensure evidence was actioned promptly when received, and stated that “ *the report was subsequently discussed in more detail and its findings have been actively incorporated into the groups' plans and recommendations to Board and to the Women and Children's Division*.”

The findings of the study were covered by UK- and US-based lay media, including in articles in the Guardian ^117^, the Independent ^118^, BBC News ^119^, the New York Times ^120^, and Propublica ^121^. The study was also cited in three articles in The Conversation (two in English ^122, 123^ and one in Spanish ^124^), and in the Indonesian- ^125^, Vietnamese- ^126^ and Ukrainian-language ^127^ versions of the Wikipedia page “Covid-19 in pregnancy”.

### Case study 2: Antenatal pulmonary embolism: risk factors, management and outcomes

The second most highly-cited UKOSS article reported surveillance of pulmonary embolism ^11^ and received 16 citations in policy and guideline documents. With the exception of one citation in a WHO-authored document that was classified as a policy document ^128^, all of these citations were in clinical guidelines, from organisations including the UK’s Royal College of Obstetricians and Gynaecologists ^129– 132^, Ireland’s Health Service Executive ^133^, the Scottish Intercollegiate Guidelines Network ^132^, the UK’s National Institute for Clinical Excellence (NICE) ^134^, the American College of Obstetricians and Gynecologists ^135, 136^, the European Resuscitation Council ^137^ and the European Society of Cardiology ^138^.

This study was among the first to be undertaken through the UKOSS platform and the article was published in February 2008. It was first cited in October 2010, in resuscitation guidelines for cardiac arrest in special circumstances from the European Resuscitation Council ^137^. The median time from publication to citation of this article was a little over 10 years (539.1 weeks). Most recently, it was cited in June 2025, over 17 years after publication, in the WHO recommendations on the management of sickle cell disease during pregnancy, childbirth and the interpregnancy period ^128^. There, it was used to provide evidence for obesity as a risk factor for pulmonary embolism.

Exploring the context of citations revealed that the study was primarily used to provide evidence about incidence, case fatality and risk factors. Several guidelines also used the study to support recommendations on management, in the form of evidence on the safety and dosing regimens, and need for monitoring, of low molecular weight heparin (LMWH). For example, the American College of Chest Physicians’ Evidence-Based Clinical Practice Guidelines on VTE, Thrombophilia, Antithrombotic Therapy, and Pregnancy Antithrombotic Therapy and Prevention of Thrombosis cite the article in support of the following guidance: “ *Observational studies confirm the safety and efficacy of LMWH in this patient population when used for treatment of VTE*… *some…recommend a bid LMWH dosing schedule. However, many clinicians use a once-daily regimen to simplify administration and enhance compliance. Observational studies have not demonstrated any increase in the risk of recurrence with the once-daily regimen over the bid regimen*” ^139^, and the European Society of Cardiology’s guidelines on the management of cardiovascular diseases during pregnancy cite the article when stating that “ *Given the need for dose increase as pregnancy progresses to maintain a certain therapeutic anti-Xa level, it seems reasonable to determine anti-Xa levels also during pregnancy in patients with VTE [venous thromboembolism]. This appears particularly justified in view of the fact that pulmonary embolism occurred in women receiving preventive doses of LMWH.*” ^138^

Altmetric did not detect any coverage of the article in the mainstream media.

### Case study 3: Uterine rupture by intended mode of delivery in the UK: a national case-control study

The third most highly-cited UKOSS article reported a case-control study of uterine rupture published in 2012 ^14^. Around half of the citations (6/13) of this article were in documents classified as clinical guidelines, published by organisations including the UK’s Royal College of Obstetricians and Gynaecologists ^140^, and several NHS Trusts ^141, 142^. The other half (7/13) were in materials classified as policy documents, which included recommendations from the national governments of Georgia ^143^ and the Republic of Slovakia ^144^, and state governments in Australia ^145, 146^.

The article was first cited around 3 months (14.1 weeks) after publication, in a “Children, families and maternity e-bulletin” issued by the UK’s Department of Health, which highlighted risk factors that should be considered when discussing and managing labour with women who have previously given birth by caesarean section ^147^. All subsequent citations occurred at least 3 years after the article was published and the most recent appeared in April 2025, thirteen years after the article was published, in an updated guideline on management of uterine rupture. The article was primarily cited as evidence of risk factors for uterine rupture, with four articles also including incidence data ^140– 142, 148^.

The study was referenced in an article published by Australian academics in The Conversation in January 2020 ^149^. Citations of the study in Australian policy documents came both before and after this article was published.

### Case study 4: Perinatal outcomes after maternal 2009/H1N1 infection: national cohort study

The joint fourth most highly cited UKOSS article reported surveillance of stillbirth, perinatal mortality, and neonatal mortality in women infected with H1N1 influenza (swine flu) during the second wave of the 2009 pandemic ^12^. This study received 12 citations in a range of documents including clinical and public health guidance and national public health plans, mainly from organisations based in the UK, the rest of Europe, and North America.

The most rapid citation detected after publication came just over a year after article publication, in the NHS Scotland’s seasonal influenza vaccination programme for 2012–13 ^150^. The median time from publication to citation was just over 4 years (221.3 weeks) and the most recent citation came in April 2021, when the study was used by the World Health Organization’s Global Advisory Committee on Vaccine Safety in a review of the evidence around the safety of vaccinations during pregnancy ^151^. There, the study was used as evidence of adverse perinatal outcomes associated with maternal pandemic influenza. Exploring the context of the twelve citations revealed that the study was in most cases cited as evidence for adverse outcomes of influenza in pregnancy and thus as a rationale for vaccinating pregnant women. For example, the Government of Canada’s statement on seasonal influenza vaccine for 2014–2015 cited the study in support of the statement, “ *NACI recommends the inclusion of all pregnant women, at any stage of pregnancy, among high priority recipients of influenza vaccine due to...evidence of adverse neonatal outcomes associated with respiratory hospitalization during pregnancy or influenza during pregnancy*” ^152^. Altmetric did not detect any coverage of the article in the mainstream media.

### Case study 5: Severe maternal sepsis in the UK, 2011–2012: A national case-control study

With the joint-fourth highest number of citations, the 2014 article reporting UKOSS surveillance of sepsis was also cited 12 times ^13^. A third of the citations were in documents classified as clinical guidance, including the American College of Obstetricians and Gynecologists’ guideline on critical care in pregnancy ^153^, and a report of the French national confidential enquiry into maternal deaths ^154^. Two thirds of the citations were in policy documents, which included the UK Chief Medical Officer’s 2014 annual report on women’s health ^155^; five World Health Organisation recommendation documents ^156– 160^; a further WHO report titled “Addressing gender inequalities in national action plans on antimicrobial resistance: guidance to complement the people-centred approach” ^161^; and a Royal College of Obstetricians and Gynaecologists document, “Care of the critically ill woman in childbirth; enhanced maternal care” ^162^.

The median delay from article publication to citation was just under 7 years (362.4 weeks), with the earliest citation occurring a little over a year after publication, in the *WHO recommendations for prevention and treatment of maternal peripartum infections* ^157^. The most recent citation came in 2024 in Ireland’s Health Protection Surveillance Centre’s “Guidelines for the Public Health Management of Contacts of Invasive Group A Streptococcus (iGAS) infection in Ireland” ^163^.

The citations appeared most frequently in the context of identifying risk factors for sepsis. Other contexts included citation as evidence of the need to treat sepsis as an emergency ^154, 163^; and the potential for physical and psychological problems during rehabilitation: “ *many critical care units assess the need for and provide support for the physical, psychological and cognitive problems that may occur during recovery and rehabilitation. The obstetric population is not immune from such problems. The psychological effects or physical complaints, such as muscle wasting, may make attending to the neonate extremely challenging*.” ^162^

The article was promoted in a press release published on EurekAlert! ^164^ Altmetric did not detect any mentions in major mainstream lay media outlets.

## Discussion

This study identified extensive, varied, and geographically widespread use of evidence generated through the UKOSS platform in policy and clinical guidelines. It also revealed a typical delay of several years or more between an article being published and it being cited in these types of documents.

We found that 65% of articles reporting primary research that used the UKOSS platform for data collection had been cited in at least one policy document or clinical guideline at the time of analysis. This citation frequency is high compared with that of published health science research in general: a 2021 analysis of Scopus-indexed healthcare research articles using Overton estimated that approximately 13% of articles published in 2008 had been cited at least once in policy documents, with a gradual decrease to approximately 6% of articles published in 2016 that probably resulted from the delay between publication and citation ^165^. Because of the relatively small number of UKOSS articles published each year, we did not analyse citation frequency by article publication year, meaning our analysis is not directly comparable with that study. However, the articles we included were published between 2007 and 2024, making the “oldest” a few years older than those in the Scopus analysis and the “youngest” considerably more recent. We would expect citations to the most recently published UKOSS articles to continue to accrue, so comparison of the two studies would, if anything, underestimate the relative uptake of UKOSS studies in policy and guidelines. Our analysis also included citations identified by only Altmetric as well as those detected by Overton, but these accounted for under a third of the citations detected and only six of the ninety-six articles received citations that were detected by Altmetric while having none detected by Overton. Thus, the data suggest comparatively high uptake of UKOSS evidence in policy and clinical guidance.

We suggest that the high uptake of UKOSS evidence reflects the fact that the UKOSS platform was designed to provide data on uncommon conditions, for which evidence to underpin policy and clinical decision-making is scarce and difficult to collect because of the small number of women affected ^1, 2^. Thus, what little robust evidence is available tends to be highly cited. This probably also explains why the earliest UKOSS articles are still occasionally being cited over 17 years after their initial publication: there is a dearth of more recent robust evidence. The high uptake rate, particularly in clinical guidelines, may also result from the choice of conditions for study being driven by clinicians and clinical researchers ^1^, meaning the findings inform areas with pressing need for evidence to underpin clinical decisions. Although UKOSS provides data on the UK pregnant population, over two thirds of citations came from organisations outside the UK. Again, this may reflect limited data available for the national population in many countries, as well as the use of UK data for comparison with that of the country in question where available. Since UKOSS was established in 2005, a number of other countries have used the same methodology to establish national and regional surveillance systems and now form INOSS, the International Network of Obstetric Surveillance Systems, with member countries including Belgium, a Nordic collaboration (Denmark, Finland, Iceland, Norway, and Sweden), France, Italy, Namibia, the Netherlands, Slovakia, Slovenia, and the UK ( https://www.npeu.ox.ac.uk/inoss/inoss-members/oss-members). As studies are conducted through these systems, the international evidence base on uncommon disorders in pregnancy will expand.

The median lag between article publication and citation was just over four years, or just under five years if the one highly and rapidly cited article reporting Covid-19 surveillance was excluded. This delay could result from a mismatch between research topics and policy priorities, or suboptimal stakeholder communication. Timelines for clinical guideline development and updating may also be a factor; for example, NICE guidelines are updated on a five-year cycle (although updates can be brought forward if sufficient new evidence warrants this) ^166^. The long median delay is also partly attributable to the fact that older studies continued to be sporadically cited long after publication; the modal delay is shorter, at around 2 years. Nonetheless, speeding up the initial uptake of evidence into policy and clinical guidelines would allow pregnant women and their babies to benefit from evidence-based practice sooner; thus, knowledge mobilisation should be prioritised for future studies. The Covid-19 pandemic was a highly atypical situation, but the speed with which evidence was integrated into policy and guidelines at this time suggests that it is possible to speed up the pipeline from research to policy and practice, given sufficient resources, aligned priorities, good communication between stakeholders, and a collective sense of urgency. 

The exploratory nature of this study and relatively small number of articles and citations involved mean we cannot draw firm conclusions about study or article characteristics that led to high numbers of citations. However, one observation worth noting is that with the exception of the study reporting Covid-19 surveillance, all of the highly cited articles profiled in our case studies reported investigations that included a comparison group: they either had a case-control design or included an external comparison group for the cohort. Among the included articles overall, this was the case for under half of the studies reported (42/96); other studies were purely descriptive or included comparisons only between subgroups within the cohort of women with the condition of interest. It is likely that the nature and strength of the evidence provided by a study with a comparison group made the highly-cited reports more useful to policymakers and guideline developers; it would be worth consulting with end-users of UKOSS evidence to ensure studies are designed with their evidence needs in mind.

One factor not captured by the methodology used in this study is potential effects on citation rates of dissemination modes other than published journal articles. Early discussions of evidence with policymakers and clinicians sometimes occurs before article publication. This is particularly relevant to two of the highly cited articles in our case studies – surveillance of 2009/H1N1 influenza and SARS-CoV-2 *.* The pandemic nature of these two conditions and the coordinated response by researchers, policymakers and medical staff meant that unpublished findings were communicated to stakeholders as soon as available. The evidence quickly fed into policy and clinical guidelines, considerably before the respective articles were published. Thus, it is possible that in some cases, high rates of citation of the eventual journal articles in policy documents result from earlier successful dissemination through other routes, meaning that the subsequent citations detected in policy documents represent a record rather than a driver of impact.

We used Altmetric to evaluate mentions of highly-cited UKOSS papers in the lay media to look for any patterns of coverage that might have facilitated their extensive uptake into policy and guidelines. The article reporting surveillance of Covid-19 in pregnancy received the most media coverage; however, given the unusually intense focus from media, policymakers, and clinical guidance developers on research relating to Covid-19 at the height of the pandemic, it is difficult to draw any generalisable conclusions around possible impact of dissemination in the lay media on uptake into policy. Altmetric did not detect any mentions in major news outlets of any of the other highly cited articles that we included in our case studies. However, this may be because of limitations in detection rather than a lack of coverage: a Google search for “swine flu perinatal maternal pregnancy 2009 Marian Knight news coverage” returned articles published by BBC News ^166^ and Sky News ^167^ that could be determined by the details included to be referring to the article on H1N1 flu surveillance ^12^. Similarly, a Google search for “sepsis perinatal maternal pregnancy 2014 Marian Knight news coverage” returned a BBC News article ^168^ that included a link to the published article. Detection by Altmetric may be problematic when a link to the study is not included in the news article, and the age of the study may also be a factor.

### Limitations

This study had several limitations. Screening of citations by a single reviewer created potential for subjectivity in inclusion and exclusion decisions. Additionally, owing to the limited resources available for the study, we limited the exploration of citation context to descriptive case studies rather than a comprehensive analysis of all citations.

The coverage of policy sources by Overton and Altmetric is extensive but not fully comprehensive; therefore, some citations were likely missed. This would include documents not published online and those posted behind a paywall. Documents in languages other than English, although not excluded by the databases, may have less comprehensive coverage, and the coverage of sources may vary between countries. Coverage of older documents is likely to be less comprehensive: Overton states that “coverage is generally good from 2015 onwards and sparse before 2009” ^166^. At the other extreme of the citation timeframe, the sharp drop-off in annual citation counts after 2021 ( Figure 4a) suggests a possible indexing delay. Additionally, the citation tracking approach to evaluating evidence use cannot detect instances where evidence was used in policy development but not cited. All of these limitations could have resulted in underascertainment of citations. The categorisation of citing organisations and type of document used the categories assigned by Overton and Altmetric, and thus is dependent on the accuracy of the information provided by the databases. Similarly, the accuracy of data such as the date of citation was dependent on Overton and Altmetric providing accurate data.

## Conclusions

Evidence from studies conducted via the UKOSS platform has been used extensively over the platform’s 20-year history in policy documents and clinical guidance in the UK and globally. Consideration should be given to how to speed up knowledge translation to allow pregnant women and their babies to benefit from evidence-based policies and practice with minimum delays.

## Ethics and consent

No ethics approval or consent was required for this retrospective analysis of publicly available documents.

## Data Availability

The data for this article includes bibliographic references (i.e., included articles), which are included in the References section. Because Overton and Altmetric are commercial databases, the underlying policy citation data cannot be shared openly due to copyright licensing restrictions. The Methods section contains detailed information to allow replication of the study using the bibliographic information of the included articles. Alternatively, requests to access the data can be made by contacting the National Perinatal Epidemiology Unit data access committee via general@npeu.ox.ac.uk and will be considered in the context of the conditions of the database licences. The estimated response time for requests is 4 weeks. For more information about procedures and conditions for data access, please refer to the National Perinatal Epidemiology Unit Data Sharing Policy available at https://www.npeu.ox.ac.uk/assets/downloads/npeu/policies/Data_Sharing_Policy.pdf.

## Software availability statement

All statistical analyses were performed using Microsoft Excel, a commercial software. All analyses in this study can be replicated using freely available statistical software such as LibreOffice Calc.
