# Supplementary material for: Use of evidence from the UK Obstetric Surveillance System in policy and clinical practice: a descriptive study using citation tracking
Source: NIHR Open Res. 2026 Jan 8;6:5. [Version 1] doi: 10.3310/nihropenres.14196.1 (PMC13156565; doi:10.3310/nihropenres.14196.1)
Supplement: Peer review history

# Reviewer report: Reviewer response for version 1

**DOI:** 10.3310/nihropenres.15453.r40423

**Published:** 2026-05-08

**Authors:** Josefine Königbauer

**Affiliations:** 1 Charité – Universitätsmedizin Berlin, Berlin, Germany

Dear authors, 

 Thank you very much for your important research.

 The manuscript addresses an important and underexplored topic by evaluating the uptake of UKOSS-derived evidence into policy documents and clinical guidelines. The study is well conceived, methodologically transparent, and provides valuable insights into the translation of maternal health research into policy and practice. The case studies are particularly informative and illustrate the breadth of influence of UKOSS evidence internationally.

 The manuscript’s main strengths are the comprehensive identification of UKOSS publications, the systematic use of Overton and Altmetric for policy citation tracking, and the thoughtful discussion of knowledge translation timelines. The findings are highly relevant for implementation science and maternal health policy research.

 However, the manuscript would benefit from several revisions.

 First, the distinction between policy citation, evidence uptake, and actual clinical or policy impact should be clarified more explicitly throughout the manuscript, as citations alone cannot demonstrate implementation or causal influence.

 Second, the paper is considerably longer than necessary, particularly in the case study sections, where several examples and quotations could be condensed without loss of content.

 Third, additional methodological clarification would strengthen reproducibility, particularly regarding citation date definitions, handling of updated guideline versions, and the implications of single-reviewer screening.

 Finally, the Discussion occasionally becomes speculative and would benefit from more cautious interpretation of the findings.

 Overall, this is a strong and potentially indexable study with clear relevance to policy translation and maternal health research. I would recommend minor revision, primarily to improve focus, analytical framing, and conciseness.

Is the work clearly and accurately presented and does it cite the current literature?

Yes

If applicable, is the statistical analysis and its interpretation appropriate?

Yes

Are all the source data underlying the results available to ensure full reproducibility?

Yes

Is the study design appropriate and is the work technically sound?

Yes

Are the conclusions drawn adequately supported by the results?

Yes

Are sufficient details of methods and analysis provided to allow replication by others?

Yes

Reviewer Expertise:

Obstretics, Quality care in obstetrics

I confirm that I have read this submission and believe that I have an appropriate level of expertise to confirm that it is of an acceptable scientific standard.

# Reviewer report: Reviewer response for version 1

**DOI:** 10.3310/nihropenres.15453.r39805

**Published:** 2026-03-23

**Authors:** Pensée Wu

**Affiliations:** 1 Keele University, Keele, England, UK

Summary

 This manuscript provides a clear description of how the evidence generated through the UK Obstetric Surveillance System (UKOSS) has made an impact in terms of policy documents and clinical guidelines over its first 20 years. It uses an interesting methodology with citation tracking in Overton and Altmetric. The authors present in-depth case studies of highly cited articles and show that around two‑thirds of UKOSS studies have informed at least one policy or guideline, with citations from national and international organisations. The citations often persist many years after publication, but citation into formal documents can lag the publication itself by several years.

Is the work clearly and accurately presented, and does it cite the current literature?

 This work is well written with a clear structure and accurate presentation throughout. It cites current literature within its methodological and policy context. The authors could consider referencing papers on research-policy translation and knowledge mobilisation, to widen the scope beyond Overton and Altmetric.

Is the study design appropriate and is the work technically sound?

 The methodology is appropriate for the aims of the study. The identification of UKOSS articles with an internal list augmented by Pubmed search is sound. The inclusion/exclusion criteria are logical and clearly described. The authors could consider stratified analyses of citations and latency by study design or topic area to provide more insight into the factors that affect impact.

Are sufficient details of methods and analysis provided to allow replication by others?

 The methods and analysis provide sufficient details to allow replication by others. 

If applicable, is the statistical analysis and its interpretation appropriate?

 The descriptive statistics used in this work are appropriate for assessing citation patterns. The statistical analysis is clearly described and correctly interpreted.

Are all the source data underlying the results available to ensure full reproducibility?

 Without contacting the National Perinatal Epidemiology Unit, full reproducibility is only partially achievable, as the list of included UKOSS articles was derived from both an internal UKOSS team list and a PubMed search. Although full reproducibility is possible by contacting the National Perinatal Epidemiology Unit, the process can take 4 weeks. Therefore, it would be helpful if a list of all included UKOSS articles could be provided in an open repository or in supplementary materials to accompany this manuscript.

Are the conclusions drawn adequately supported by the results?

 The results support the conclusions that UKOSS-derived evidence is widely used in policy and guidance, that citations are geographically diverse but focused in high‑income settings similar to the UK, and that the median citation latency is around four years. The case studies highlight the variety of citation contexts and their impact on policy or practice. The discussion fully addressed the limitations of the study.

Is the work clearly and accurately presented and does it cite the current literature?

Yes

If applicable, is the statistical analysis and its interpretation appropriate?

Yes

Are all the source data underlying the results available to ensure full reproducibility?

Yes

Is the study design appropriate and is the work technically sound?

Yes

Are the conclusions drawn adequately supported by the results?

Yes

Are sufficient details of methods and analysis provided to allow replication by others?

Yes

Reviewer Expertise:

High risk obstetrics, maternal medicine

I confirm that I have read this submission and believe that I have an appropriate level of expertise to confirm that it is of an acceptable scientific standard.
